# LncRNAs expression profile in a family household cluster of COVID‐19 patients

**DOI:** 10.1111/jcmm.18226

**Published:** 2024-03-19

**Authors:** Iulia Virginia Iancu, Carmen Cristina Diaconu, Adriana Plesa, Alina Fudulu, Adrian Albulescu, Ana Iulia Neagu, Ioana Madalina Pitica, Laura Denisa Dragu, Coralia Bleotu, Mihaela Chivu‐Economescu, Lilia Matei, Cristina Mambet, Saviana Nedeianu, Corneliu Petru Popescu, Camelia Sultana, Simona Maria Ruta, Anca Botezatu

**Affiliations:** ^1^ Stefan S Nicolau Institute of Virology Bucharest Romania; ^2^ Department of Pharmacology National Institute for Chemical Pharmaceutical Research and Development Bucharest Romania; ^3^ Carol Davila University of Medicine and Pharmacy Bucharest Romania; ^4^ Dr Victor Babes Infectious and Tropical Diseases Clinical Hospital Bucharest Romania

**Keywords:** COVID‐19, family cluster, lncRNAs, nasopharyngeal swabs, SARS‐CoV‐2

## Abstract

More than 3 years after the start of SARS‐CoV‐2 pandemic, the molecular mechanisms behind the viral pathogenesis are still not completely understood. Long non‐coding RNAs (lncRNAs), well‐known players in viral infections, can represent prime candidates for patients' risk stratification. The purpose of the current study was to investigate the lncRNA profile in a family cluster of COVID‐19 cases with different disease progression, during the initial wave of the pandemic and to evaluate their potential as biomarkers for COVID‐19 evolution. LncRNA expression was investigated in nasopharyngeal swabs routinely collected for diagnosis. Distinct expression patterns of five lncRNAs (HOTAIR, HOTAIRM1, TMEVPG1, NDM29 and snaR) were identified in all the investigated cases, and they were associated with disease severity. Additionally, a significant increase in the expression of GAS5‐family and ZFAS1 lncRNAs, which target factors involved in the inflammatory response, was observed in the sample collected from the patient with the most severe disease progression. An lncRNA prognostic signature was defined, opening up novel research avenues in understanding the interactions between lncRNAs and SARS‐CoV‐2.

## 1 INTRODUCTION

The COVID‐19 pandemic has presented with different clinical forms, ranging from asymptomatic infections to highly severe cases with complications such as acute respiratory distress requiring invasive mechanical ventilation, coagulopathy with small vessels thrombosis, acute cytokine storm, and the potential for multi‐organ involvement and death.^1^, ^2^ Rarely, children and adults can develop a multisystem inflammatory syndrome, weeks after infection.^3^ The reported mortality rates vary between different countries and regions, and are influenced by host characteristics, viral variants and multiple social, political and administrative factors (capacity to perform tests, protocols for contact tracing, medical healthcare system and different policies regarding the calculation of deaths).^4^

The molecular mechanisms of human coronaviruses (SARS‐CoV, MERS‐CoV and SARS‐CoV‐2) that produce potentially severe symptoms are complex, involving the blocking or delaying host interferon production.^5^, ^6^ Instead, SARS‐CoV‐2 can trigger an uncontrolled response of the innate immune system, leading to a release of a large amount of chemokines and pro‐inflammatory cytokines by immune effector cells. The resultant is a phenomenon is commonly referred to as a ‘cytokine storm’ and is strongly associated with lung injury, multi‐organ failure and an increased fatality rate in severe cases of COVID‐19.^7^, ^8^, ^9^

Moreover, other studies showed that the virus may cause viral sepsis^10^ and infection can lead to deregulation in blood coagulation,^11^, ^12^ COVID‐19 being primarily a vascular, rather than a pure respiratory disease.^13^

However, additional studies are needed to decipher the mechanisms that underlie the pathogenesis of infection, its progression and the long‐term consequences.

Non‐coding RNAs (ncRNAs) have important regulatory function both at transcriptional and post‐transcriptional levels.^14^, ^15^ LncRNAs, a class of ncRNAs with lengths exceeding 200 nucleotides, are involved in various biological regulatory processes, such as inflammation, cellular functions, and in the pathogenesis of various diseases, including viral infections and immune disorders.^16^, ^17^, ^18^ Recent research suggests that lncRNAs are essential regulators of acute inflammatory reaction and thrombotic events in COVID‐19 patients, hence maintaining persistent viral infections.^19^

To date, two important lncRNAs, MALAT1 (lncRNA metastasis‐associated lung adenocarcinoma transcript 1) and NEAT1 (nuclear paraspeckle assembly transcript 1), were associated with the antiviral defence response, innate immune response and inflammatory response in SARS‐CoV‐2 infection.^20^, ^21^

In a murine model of SARS‐CoV infection, 5329 differentially expressed lncRNAs were found; some of them downregulated (e.g., MALAT1) and negatively correlated with KDELR3 (reticulum protein retention receptor 3) and TubA1A (tubulin1) genes, while other were upregulated (e.g., NEAT1) after viral infection.^22^ In COVID‐19 patients, abnormal expressions of various lncRNAs were found in bronchoalveolar lavage fluid (BALF) and peripheral blood mononuclear cells (PBMC).^20^

Differences in lncRNA gene expression profiles according to COVID‐19 evolution were also reported^23^ with lncRNA GATA5 significantly increased in severe COVID‐19 patients, and lncRNA DANCR and NEAT1 differently expressed in mild and severe COVID‐19 cases.^24^

A strong correlation between high expression levels of NEAT1 and TUG1 and the cytokine storm in moderate and severe cases were also highlighted.^25^

Recently, the HULC lncRNA overexpression has been suggested as a potential important predictor of disease severity, being associated with higher mortality rates in patients with COVID‐19.^26^

The approach of the present study aims to investigate an unexplored area of research concerning lncRNA profiling in a cluster of COVID‐19 cases within the same family. All individuals in the family were infected with one of the early variants of SARS‐CoV‐2 (PANGO B.1 lineage), during the initial wave of the pandemic. Revealing the lncRNA profiles within a family cluster of COVID‐19 patients might offer novel insights into the understanding of how epigenetic factors influence the susceptibility, severity and progression of the disease. It could pave the way for innovative therapeutic strategies and the development of personalized treatment approaches. Nevertheless, it is important to acknowledge that differences in disease severity and progression among patients within the cluster cannot be solely attributed to epigenetic factors, despite their shared backgrounds.

## 2 MATERIALS AND METHODS

### 2.1 Biological specimens

Nasopharyngeal swabs were collected from four COVID‐19 patients (members of the same family: parents and their offspring) who tested positive for SARS‐CoV‐2 infection in December 2020. The swabs were collected within few hours, with a maximum timeframe of 1 day from the onset of symptoms. Among these patients, two subjects required hospitalization (one severe form at admission and the other moderate form). In addition to the positive samples, SARS‐CoV‐2 negative, control samples, collected from the same individuals prior to their infection during routine testing were also available. Written informed consent was obtained from all investigated subjects. The study protocol, including the informed consent procedure, was approved by the institutional research ethics committee (No. 2249/25.11.2020).

### 2.2 SARS‐CoV‐2 molecular detection

Viral RNA isolation from biological specimens was initially employed for SARS‐CoV‐2 molecular detection. SARS‐CoV‐2 nucleic acid extraction was carried out using the *QIAmp Viral Mini Kit* (Qiagen, Germany) following the manufacturer's instructions. Subsequently, a TaqMan‐based RT‐PCR assay was performed on investigated samples using *VIASURE SARS*‐*CoV*‐*2 Real Time PCR Detection kit* (CerTest Biotec, Spain). The assay targets ORF1ab and nucleocapsid (N) genes from the SARS‐CoV‐2 viral genome.

### 2.3 SARS‐CoV‐2 genome characterization

SARS‐CoV‐2 variants identification in patient's samples was done by virus whole‐genome sequencing on MiSeq system (Illumina, USA) using Total RNA library preparation with *Ribo‐Zero Plus rRNA Depletion* workflow and according to the manufacturer's specifications. Subsequently, data analysis was done using a BaseSpace pipeline (Illumina, USA) namely DRAGEN COVID Lineage App (v3.5.7) and for clade/lineage classification Pangolin and NextClade methods were used. The sequences have been submitted and are available at the open‐access platform and database for researchers and public health officials: Global Initiative on Sharing All Influenza Data (GISAID‐https://www.epicov.org/epi3/frontend#598ecf) with the following accession IDs: EPI_ISL_1081954; EPI_ISL_1260862; EPI_ISL_1260871; EPI_ISL_1265373.

### 2.4 LncRNAs profiling

Total RNA was isolated from biological samples using *TRIzol* reagent (Thermo Fisher Scientific, USA) and purified using *RNeasy Kit* (Qiagen, Germany). The expression profile of lncRNAs in patient's nasopharyngeal swabs was evaluated using *LncRNA Profiler qPCR Array Kit* (System Biosciences, SBI, USA), a SYBR Green qPCR assay that evaluates 90 human lncRNAs and five house‐keeping genes expression levels (18S rRNA, RNU43, snRNA U6B, GAPDH and Lamin A/C) on an ABI 7300 Real Time System (Applied Biosystems, ABI, USA). The investigation was performed in triplicate, and the results are presented as normalized lncRNA expression levels (fold change) after $2^{\left(-{\Delta \Delta C}_{q}\right)}$ analysis using double normalization method. The cut‐off value for n‐fold was established at >2. The expression levels of lncRNAs isolated from control samples were used in the double normalization formula.

## 3 RESULTS

### 3.1 Clinical cases presentation

The four patients presented distinct clinical manifestations (Table 1). Among them, the eldest patient, aged 71 years, had a number of comorbidities (primary hypertension under treatment; stroke motor sequelae) and displayed the most severe form of the disease. This patient exhibited severe lymphopenia, increased CRP (14 mg/dL), hyperglycaemia and high levels of serum D‐dimers (>20 μg/mL). The chest X‐ray examination of this patient (1) revealed ground‐glass opacities in the right infrahilar and infiltrate in the left paracardiac area.

**TABLE 1:** Clinical and epidemiological data of the SARS‐CoV‐2 positive patients belonging to the family cluster.

Patient no	1	2	3	4
Age (years)	71	66	35	31
Gender	Male	Female	Female	Male
Patient medical history	Primary hypertension under treatment; stroke motor sequelae (2014)	Primary hypertension under treatment; insulin‐dependent type II diabetes; mild secondary thrombocytopenia	None	None
Symptoms/Clinical manifestations				
Fever	x	–	–	x
Myalgia	x	x	x	x
Malaise	x	x	x	x
Sore throat	x	x	x	x
Cough	x	x	–	x
Headache	x	x	x	x
Loss of taste and smell	–	x	x	x
Digestive manifestations (nausea/ vomiting/ diarrhoea)	–	–	–	–
Shortness of breath	x	–	–	–
Dyspnoea	x	–	–	–
Abnormal chest imaging	x	–	–	–
Oxygen saturation in the ambient air (SpO_2_%) <94%	x	–	–	–
Lung infiltrates (>50%)	x	–	–	–
Diagnosis	Bilateral interstitial pneumonia; SARS‐CoV‐2 severe form infection	Acute interstitial pneumonia; SARS‐CoV‐2 mild form infection	Acute interstitial pneumonia; SARS‐CoV‐2 moderate form infection	Acute interstitial pneumonia; SARS‐CoV‐2 moderate form infection
Biochemical parameters				
White blood cells WBC (/μL)	7600	5300	4600	7500
Neutrophils (N %)	**71.9**	60.9	**36.4**	66.8
Lymphocyte (L %)	**20.2**	27.7	**50.5**	**23.9**
NLR ratio	**4**	2	1	3
Platelets PLT (/μL)	269,000	**135,000**	285,000	196,000
Haemoglobin Hb (g/dL)	15.1	15	**11.7**	14.9
C‐reactive protein CRP (mg/dL)	**2.55**	**0.78**	0.18	**1.48**
D‐dimers (μg/mL)	**0.57**	0.24	**0.54**	0.24
Fibrinogen (mg/dL)	**522**	**412**	373	381
Ferritin (ng/mL)	243.6	201.2	23.3	**386.6**
Prothrombin ratio (PR)	88	110	90	91
International normalized ratio (INR)	1.09	0.94	0.87	1.07
Quick time (sec)	14.5	12.6	11.7	14.2
ALT (UI/L)	**70**	46	21	29
LDH (UI/L)	227	**104**	169	116
Glucose (mg/dL)	100	**204**	99	89
Creatinine (mg/dL)	0.9	0.6	**0.5**	1.1
Hospitalized	Yes	Yes	No	No

*Note*: X, present; −, absent.

Values highlighted in bold are out of the reference range.

Patient 1 received treatment with remdesivir, dexamethasone and anticoagulants, together with oxygen supplementation for 26 days. After 32 days, patient was discharged with a radiologic reduction in interstitial alveolar foci and oxygen saturation of 95%.

The second elderly patient (female, aged 66 yrs) experienced a mild form of infection with a favourable evolution after dexamethasone administration and she was discharged after 10 days.

The other two family members (female aged 35 yrs and male aged 31 yrs) presented moderately symptomatic forms of infection, without pulmonary complications and they did not require hospitalization.

### 3.2 SARS‐CoV‐2 variants identification

The phylogenetic analysis of the complete genome sequencing of SARS‐CoV‐2 revealed the presence of the PANGO B.1 lineage, G clade, one of the circulating variants in December 2020 in Romania, this variant being identified in all patients (GISAID accession number: EPI_ISL_1081954; EPI_ISL_1260862; EPI_ISL_1260871; and EPI_ISL_1265373).

### 3.3 LncRNA expression profile

A distinct lncRNA expression profile was observed based on the severity of the disease. An n‐fold>2 was established as a cut‐off value for significant expression level modifications, and the results are illustrated as log10 fold change. Among the 40 lncRNAs exhibiting significantly modified expression levels, 39 were found to be overexpressed at high levels in the patient with the most severe form of the disease. These lncRNAs include IGF2AS, NcR‐UPAR, EgoA, ZFAS1, LUST, Zfhx2as, HOXA11S, GAS5‐family, 21A and NDM29.

**FIGURE 1:**
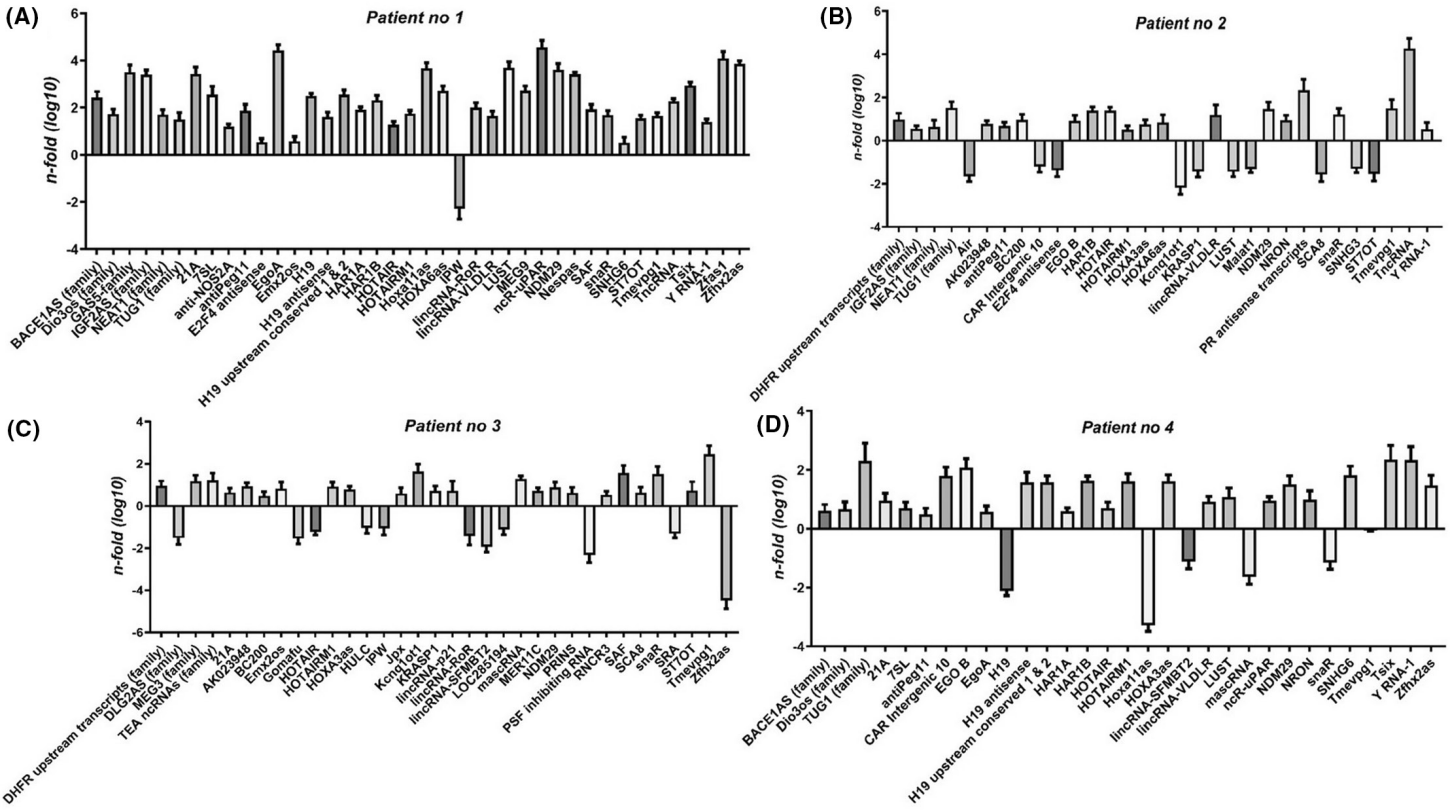
LncRNA expression profiles in COVID‐19 patients. (A) Aberrant lncRNA expression profile in COVID‐19 severe form. (B) LncRNA expression profile in COVID‐19 mild form. (C, D) LncRNA expression patterns in COVID‐19 moderate cases.

NcR‐UPAR (non‐coding RNA upstream of the PAR‐1) has the highest overexpressed level (log10 n‐fold = 4.54), while IPW (imprinted in Prader–Willi syndrome, non‐protein Coding) lncRNA (log10 n‐fold = −2.28) was significantly downregulated (Figure 1A).

In the second hospitalized patient, the profiling of lncRNAs revealed a significant change in expression for 31 of the investigated targets. Among these, 10 were downregulated, with KCNQ1OT1 (KCNQ1 opposite strand/antisense transcript 1) displaying the lowest value (log10 n‐fold = −2.16), while TncRNA / NEAT1 lncRNA showed the highest value (log10 n‐fold = 4.32) (Figure 1B).

Distinct individual expression profiles were observed in the two younger patients with moderate forms of infection (Figure 1C,D).

### 3.4 Analysis of differentially expressed lncRNAs in COVID‐19 patients

**FIGURE 2:**
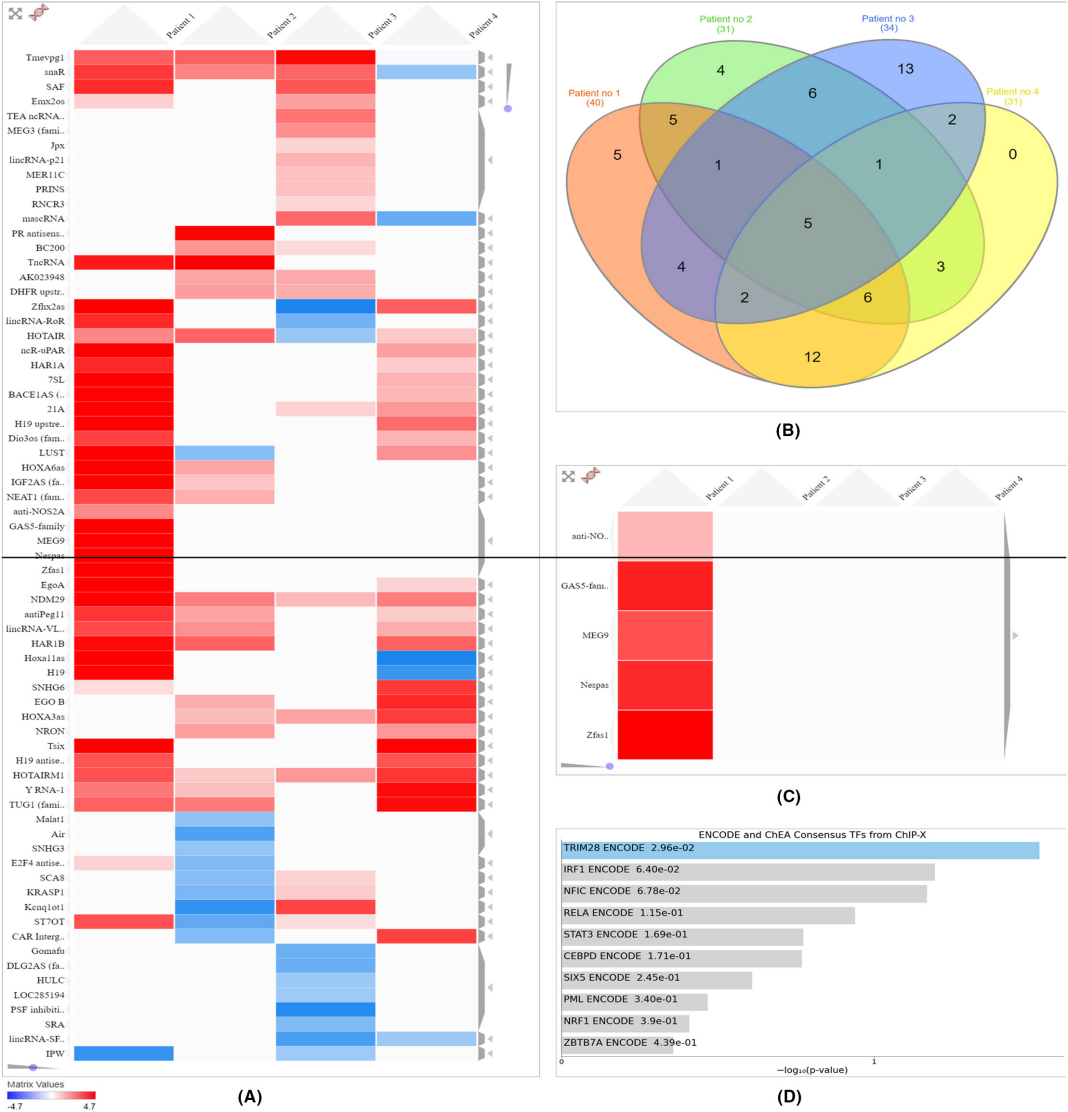
Analysis of differentially expressed lncRNAs in COVID‐19 patients. (A) A hierarchical clustering heatmap representing the expression patterns of differentially expressed lncRNAs in the investigated samples. In the heatmap representation, values show the expression levels (fold change) of lncRNAs for each patient, with red indicating high expression and blue indicating low expression. (B) Venn diagram illustrates the number of both overlapping and distinct sets of differential lncRNAs in COVID‐19 patients. (C) A distinct cluster, consisting of five lncRNA transcripts exclusively expressed in severe cases, with their upregulation highlighted in red boxes. (D) Enrichment analysis, using the Enrich tool, focusing on a subset of lncRNAs within the identified cluster that is exclusively expressed in severe case. Bar chart illustrating the highest enriched terms extracted from the gene set library ENCODE_and_ChEA_Consensus_TFs_from_ChIP‐X. The top 10 enriched terms for the subset of lncRNAs are presented, ranked by −log10 (*p*‐value), with the corresponding *p*‐value displayed alongside each term. The term positioned at the top indicates the most significant overlap with lncRNA set.^28^

Next, a comprehensive analysis was conducted using the expression profiles of lncRNAs from COVID‐19 patients, involving hierarchical clustering heatmap analysis. The heatmap was built using the Clustergrammer analysis tool,^27^ and it reveals all differentially expressed lncRNAs for the four investigated patients (Figure 2A).

A Venn diagram analysis^29^ was used to illustrate the overlapping and nonoverlapping lncRNAs significantly expressed in COVID‐19 patients (Figure 2B). Five lncRNAs are commonly dysregulated in all four patients: two chromatin regulators, HOTAIR (HOX transcript antisense RNA) and HOTAIRM1 (HOXA Transcript Antisense RNA, Myeloid‐Specific 1), TMEVPG1 (IFNG‐AS1/IFNG antisense RNA 1; NEST), an interferon modulator, NDM29 (neuroblastoma differentiation marker 29), and snaR (small NF90‐associated RNA; SNAR‐A1: small NF90 (ILF3) associated RNA).

Furthermore, through clustering analysis, a unique cluster that is only present in the patient with a severe form has been identified. This cluster is characterized by the upregulation of specific lncRNAs: anti‐NOS2A, GAS5‐family, MEG9, NESPAS/GNAS‐AS1 (GNAS Antisense RNA 1) and ZFAS1/ZNFX1‐AS1 (ZNFX1 antisense RNA 1) (Figure 2C).

In the moderate SARS‐CoV‐2 infection cases, two specific lncRNAs were detected Lnc‐SFMBT2‐3 (NONHSAG005163.2) and MASCRNA (MALAT1‐associated small cytoplasmic RNA). LncRNAs AIR/AIRN (antisense of IGF2R non‐protein coding RNA)/IGF2RAS, MALAT1, SNHG3 (small nucleolar RNA host gene 3) and PR antisense transcripts appear to be significantly expressed only in the mild case.

### 3.5 Potential molecular targets for the aberrant expressed lncRNAs

Two of the highly overexpressed lncRNAs, namely GAS5‐family and ZFAS1, were detected only in the samples of the patient with the most severe evolution. Integration into the cellular signalling pathways was considered for lncRNA whose expression pattern displayed significant changes.

**FIGURE 3:**
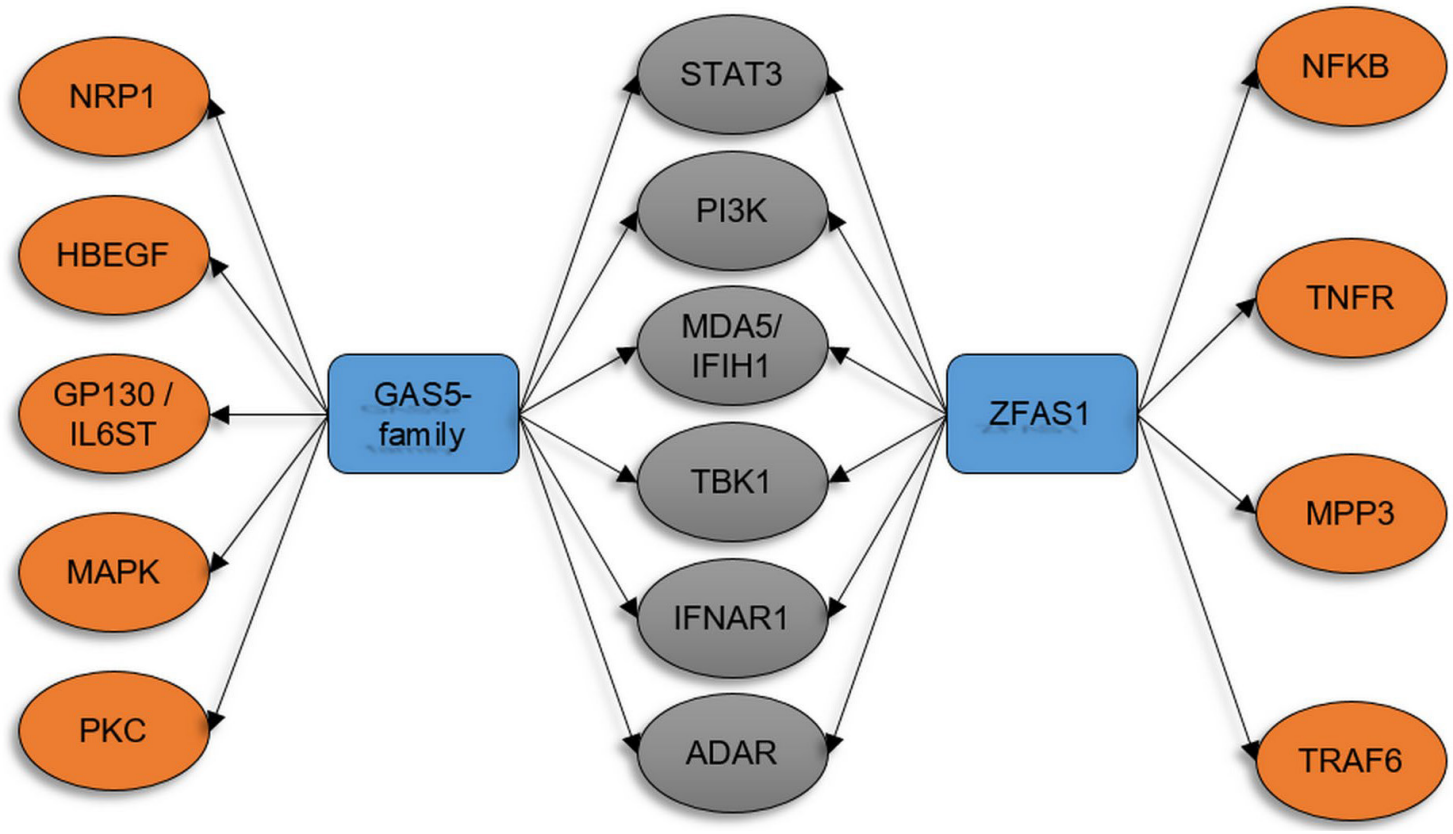
GAS5‐family and ZFAS1 lncRNAs interactions in COVID‐19. ADAR, adenosine deaminase RNA specific; GP130/IL6ST, interleukin 6 cytokine family signal transducer; HBEGF, heparin‐ binding EGF like growth factor; IFNAR1, interferon alpha and beta receptor subunit 1; MAPK, mitogen‐activated protein kinase signalling; MDA5/IFIH1, interferon induced with helicase C domain 1 encoding MDA5; MPP3, matrix metallopeptidase 3; NFKB, nuclear factor kappa B signalling; NRP1, neuropilin 1; PI3K, phosphoinositide 3‐kinase signalling; PKC, protein kinase C signalling; STAT3, signal transducer and activator of transcription 3; TBK1, TANK binding kinase 1; TNFR, TNF receptor; TRAF6, TNF receptor‐ associated factor 6.

Investigating the genes/signalling pathways targeted by these lncRNAs using a specialized database—NcPath—used for enrichment analysis and illustration of ncRNAs involvement in various KEGG signalling pathways,^30^ common cellular targets, and specifically targeted pathways (e.g., IL‐6 signalling; RIG‐I‐like receptor signalling pathway; and antiviral innate immune signalling pathways) were identified and are illustrated in Figure 3.

## 4 DISCUSSION

In this study, an elevated expression of GAS5‐family and ZFAS1, both targeting STAT3, was specifically observed in the severe COVID‐19 case. This finding may provide a partial explanation for the patient's symptoms and progression of the disease.

LncRNAs are believed to play a significant role in viral infections by targeting and modulating crucial viral sensors. Through pathway analysis, we identified that GAS5‐family and ZFAS1, two lncRNAs of interest, exhibit shared cellular targets, the majority of which are involved in the host's antiviral response. These targets include STAT3, which is a key regulator of the host's innate response to viral infections, IFNAR1, a vital antiviral factor,^31^ MDA5, an RNA antiviral sensor,^32^ and protein kinase TBK1, which participates in the induction of type I interferons in response to viral replication.^33^ STAT3 pathway involvement in SARS‐CoV‐2 infection is well documented. Several reports have demonstrated that STAT3 activation can promote and sustain COVID‐19‐associated hyperinflammatory syndrome, thrombosis, lymphopenia and lung fibrosis.^34^ An important characteristic in individuals with severe manifestations of COVID‐19 is the potential progression to acute respiratory distress syndrome (ARDS), and this is frequently associated with the presence of increased levels of various immunological factors that promote inflammation. At the core of these processes that contribute to inflammation are the activation of multiple diverse immune pathways. A notable example is IL‐6 signalling pathway, where interaction between IL‐6 and IL‐6R initiates a complex formation with the membrane protein glycoprotein 130 (gp130) and subsequently leads to the activation of the JAK/STAT pathway accompanied by STAT3 activation via phosphorylation. Also, through the PI3K‐Akt pathway, the NF‐κB factor is activated, which, alongside STAT3 activation, promotes immune cell differentiation and the expression of IL‐6 and thus inflammation.^35^ Several lncRNAs have been shown to interact and regulate the production of these inflammatory factors, one example being IL‐6 in which case its expression is controlled by different lncRNAs targeting factors involved in either or both NF‐κB and JAK/STAT pathways.^36^

There are reports indicating that ZFAS1 lncRNA is implicated in antiviral responses. It has been observed to be overexpressed in response to both RNA and DNA viruses, along with type I interferons (IFN‐I), in a Jak–STAT signalling‐dependent way.^37^ Moreover, it has been revealed that ZFAS1 exert an inhibitory effect on STAT3 as is shown in triple‐negative breast cancer patients.^38^

Concerning GAS5 lncRNA, there is evidence suggesting that its elevated expression levels may induce STAT3 degradation via ubiquitination, leading to decreased RORγt levels and consequently inhibiting TH17 differentiation in individuals with COVID‐19.^39^

Many of the immune‐relevant transcripts targeted by GAS5‐family and ZFAS1 lncRNAs in this study play significant roles in the activation of IL‐6 signalling pathway and consequently might be involved in associated regulatory pathways that orchestrating immune responses.

Thus, the increased expression of GAS5‐family and ZFAS1 targeting STAT3 may have implications for the pathogenesis and clinical outcomes of COVID‐19 cases.

In the most severe case, which was complicated by coagulation‐related issues, one of the significantly overexpressed lncRNA was NcR‐UPAR. This particular non‐coding RNA is located nearby PAR1 gene (coagulation factor II thrombin receptor) and described to upregulate its expression.^40^ The high expression of this lncRNA from the infection onset suggests its potential involvement in coagulation disturbances. Furthermore, it could serve as a potential prognostic marker for predicting the development of coagulation‐related complications in severe cases of COVID‐19.

To the best of our knowledge, this study is one of the first reports to investigate the effect of the same SARS‐CoV‐2 variant in persons with similar genetic backgrounds. This unique approach facilitated the identification of distinct or shared lncRNA expression profiles associated with disease severity. Nasopharyngeal swabs were utilized as the sample source for assessing lncRNA expression, as they provide a readily accessible specimen for the identification of novel biomarkers. According to the investigation conducted by Rodrigues A.C. et al. (2021), high levels of expression for lncRNAs NEAT1 and MALAT1 were detected in the saliva and nasopharyngeal swabs of SARS‐CoV‐2 infected patients.^41^ Our study supports these findings, highlighting elevated levels of NEAT1‐family and TncRNA lncRNAs identified in hospitalized patients.

In the moderate and severe cases of COVID‐19 investigated in this study, we were able to identify significant lncRNA expression patterns at the site of viral entry. This provided us with a snapshot of the local lncRNA profile at the onset of infection, which holds potential value for predicting disease prognosis.

These observations emphasize the potential involvement of lncRNAs in the progression of the infection in the upper respiratory tract. By gaining insights into the early molecular events associated with COVID‐19 severity and have laid the foundation for potential prognostic markers.

This study has several limitations including the small number of investigated patients, the treatment for chronic diseases in elderly patients, the lack of data for a comparative study in patients' blood samples although, lncRNA expression profile in blood samples could be more influenced by drug treatments.

Further investigations are required to better understand and to establish the precise role of lncRNAs in SARS‐CoV‐2 infection. It would be valuable to expand the study by increasing the number of patients and examining how the identified lncRNA profiles, which have diagnostic and prognostic potential, may vary in the context of various SARS‐CoV‐2 circulating variants. This opens up novel opportunities for stratifying COVID‐19 patients based on their risk profiles at the onset of the disease.

## 5 CONCLUSIONS

For COVID‐19, the growing data suggest that lncRNAs are key factors in many processes associated with the viral infection.

This study presents a specific lncRNA expression pattern associated with SARS‐CoV‐2 infection, and notably, this pattern varies depending on the severity of the infection. An increased expression of GAS5‐family and ZFAS1, both targeting STAT3 pathway, suggests that the lncRNA profile is targeting genes or pathways involved in the inflammatory response, predicting the disease severity.

Data reported in this study reveal that lncRNA profile holds promise for advancing our understanding, and by extending the numbers of samples tested, it could potentially lead to the improvement of prognostic strategies in the management of COVID‐19, by elucidating the mechanisms involved in the severe forms of the disease.

Further exploration of lncRNAs and their roles in the disease progression is warranted to shed light unto the cellular processes that made SARS‐CoV‐2 such a prolific virus and help combat the ongoing circulating variants by revealing possible targets for molecular therapy in personalized medicine approaches.

## AUTHOR CONTRIBUTIONS

**Iulia Virginia Iancu:** Conceptualization (equal); data curation (equal); formal analysis (equal); investigation (lead); methodology (equal); writing – original draft (lead); writing – review and editing (equal). **Carmen Cristina Diaconu:** Conceptualization (equal); funding acquisition (equal); methodology (equal); project administration (lead); supervision (equal); validation (equal); writing – original draft (equal); writing – review and editing (equal). **Adriana Plesa:** Data curation (equal); formal analysis (equal); investigation (equal); methodology (equal); writing – review and editing (equal). **Alina Fudulu:** Data curation (equal); formal analysis (equal); investigation (equal); methodology (equal); writing – review and editing (equal). **Adrian Albulescu:** Data curation (equal); formal analysis (equal); investigation (equal); methodology (equal); writing – review and editing (equal). **Ana Iulia Neagu:** Data curation (equal); formal analysis (equal); investigation (equal); methodology (equal); writing – review and editing (equal). **Ioana Madalina Pitica:** Data curation (equal); formal analysis (equal); investigation (equal); methodology (equal); writing – review and editing (equal). **Laura Denisa Dragu:** Data curation (equal); formal analysis (equal); investigation (equal); methodology (equal); writing – review and editing (equal). **Coralia Bleotu:** Data curation (equal); formal analysis (equal); investigation (equal); methodology (equal); writing – review and editing (equal). **Mihaela Chivu‐Economescu:** Data curation (equal); formal analysis (equal); investigation (equal); methodology (equal); writing – review and editing (equal). **Lilia Matei:** Data curation (equal); formal analysis (equal); investigation (equal); methodology (equal); writing – review and editing (equal). **Cristina Mambet:** Data curation (equal); formal analysis (equal); investigation (equal); methodology (equal); writing – review and editing (equal). **Saviana Nedeianu:** Data curation (equal); formal analysis (equal); investigation (equal); methodology (equal); writing – review and editing (equal). **Corneliu Petru Popescu:** Data curation (equal); formal analysis (equal); investigation (equal); methodology (equal); writing – review and editing (equal). **Camelia Sultana:** Data curation (equal); formal analysis (equal); investigation (equal); methodology (equal); writing – review and editing (equal). **Simona Maria Ruta:** Conceptualization (equal); supervision (equal); validation (equal); writing – original draft (equal); writing – review and editing (equal). **Anca Botezatu:** Conceptualization (equal); data curation (equal); formal analysis (equal); funding acquisition (equal); investigation (equal); methodology (equal); supervision (equal); writing – original draft (equal); writing – review and editing (equal).

## CONFLICT OF INTEREST STATEMENT

The authors declare no conflict of interest.

## Data Availability

The data that support the findings of this study are available from the corresponding author upon reasonable request.
